# Self-Reported Risk and Delinquent Behavior and Problem Behavioral Intention in Hong Kong Adolescents: The Role of Moral Competence and Spirituality

**DOI:** 10.3389/fpsyg.2018.00430

**Published:** 2018-03-29

**Authors:** Daniel T. L. Shek, Xiaoqin Zhu

**Affiliations:** ^1^Department of Applied Social Sciences, The Hong Kong Polytechnic University, Kowloon, Hong Kong; ^2^Centre for Innovation Programmes for Adolescents and Families, The Hong Kong Polytechnic University, Kowloon, Hong Kong; ^3^Department of Social Work, East China Normal University, Shanghai, China; ^4^Kiang Wu Nursing College of Macau, Macau, China; ^5^Hong Kong Institute of Service Leadership & Management Limited, Wanchai, Hong Kong; ^6^Division of Adolescent Medicine, Kentucky Children’s Hospital, University of Kentucky, Lexington, KY, United States

**Keywords:** delinquent behavior, longitudinal study, developmental trajectory, developmental asset, Chinese adolescents

## Abstract

Based on the six-wave data collected from Grade 7 to Grade 12 students (*N* = 3,328 at Wave 1), this pioneer study examined the development of problem behaviors (risk and delinquent behavior and problem behavioral intention) and the predictors (moral competence and spirituality) among adolescents in Hong Kong. Individual growth curve models revealed that while risk and delinquent behavior accelerated and then slowed down in the high school years, adolescent problem behavioral intention slightly accelerated over time. After controlling the background socio-demographic factors, moral competence and spirituality were negatively associated with risk and delinquent behavior as well as problem behavioral intention across all waves as predicted. Regarding the rate of change in the outcome measures, while the initial level of spirituality was positively linked to the growth rate of risk and delinquent behavior, the initial level of moral competence was negatively associated with the growth rate of problem behavioral intention. The theoretical and practical implications of the present findings are discussed with reference to the role of moral competence and spirituality in the development of adolescent problem behavior.

## Introduction

Adolescence is portrayed as a life stage full of “storm and stress.” While most adolescents can successfully adapt to adolescent challenges, some young people have maladaptive problems (Cheung, 2014; Tsitsika et al., 2015; Calado et al., 2017). Adolescent problem behaviors not only hinder adolescents’ current healthy functioning but also adversely impact their well-being in the long run. For instance, adolescents with higher probability of engaging in fighting and petty theft were more likely to report depression, substance abuse problems and dropping out of high school in early adulthood (Cook et al., 2015). A review based on studies published over a 25-year span showed that adolescents using alcohol, tobacco and other drugs frequently performed more badly academically, completed fewer years of education and were less likely to attend colleges compared with their peers without substance abuse problems (Bradley and Greene, 2013).

As adolescent problem behaviors have high personal, family and social costs, there is an urgent need to prevent adolescent problem behaviors. As some researchers argued that some internal assets could act as protective factors to reduce the occurrence of adolescent problem behaviors (Lo et al., 2011), the present study focused on two developmental assets (moral competence and spirituality) and examined how they predicted the externalizing behaviors indicated by risk and delinquent behavior and the intentions to engage in problem behaviors in Hong Kong adolescents.

### Positive Youth Development and Externalizing Problem Behaviors

Positive youth development (PYD) attributes are well recognized as protective factors of adolescent externalizing problems (Lerner et al., 2013; Shek and Leung, 2013). The PYD perspective holds that multiple developmental assets help to buffer the negative influence of life difficulties during adolescence, which prevents adolescents from involving in problem behaviors (Lerner et al., 2013). For instance, in Geldhof et al.’s (2014) longitudinal study involving American students from Grade 5–12 (i.e., eight waves), PYD assessed as five Cs (i.e., competence, confidence, connection, character and caring) was inversely related to delinquency and substance use in all waves. Likewise, in a large-scale study which defined PYD in terms of 15 developmental assets (e.g., resilience, behavioral competence, moral competence, emotional competence, and spirituality), Shek and collaborators found that PYD had inverse concurrent and longitudinal associations with externalizing problem behaviors among Chinese adolescents (Sun and Shek, 2012; Shek and Lin, 2016a,b).

Besides, there are findings showing the linkage between problem behaviors and specific developmental assets such as moral competence and spirituality (e.g., Salas-Wright et al., 2013; Hardy et al., 2014), both of which are important elements of the 15 PYD constructs highlighted by Catalano et al. (2002). Unfortunately, most of the existing studies are based on cross-sectional data obtained in the Western societies. Although common psychosocial correlates (e.g., self-control) were found for delinquency in the Western and Chinese cultures (Chui and Chan, 2016), cultural differences were also observed with respect to adolescent perceptions and evaluations of delinquent behaviors (Tisak et al., 2017). Therefore, much remains unknown regarding the effect of a single developmental asset on adolescent externalizing problems in the Chinese societies.

Regarding the development of problem behaviors such as delinquency, the research findings in the Western and Chinese contexts suggested an inverted U-shaped trajectory featured with an increase in early adolescence and a decline in early adulthood (Deković et al., 2004; Shek and Lin, 2016a). However, relatively fewer studies attempted to uncover the relationship between the PYD attributes and the rate of change in adolescent problem behaviors. Among those who did, Shek and Lin (2016a,b) investigated how initial general PYD attributes encompassing the 15 developmental assets predicted the growth rate of delinquency over time. However, no research has to date been conducted to examine how specific developmental assets are related to the growth rate of risk and delinquent behavior and problem behavioral intention. To fill this gap, the present study focused on two specific developmental assets, namely moral competence and spirituality.

### Moral Competence and Externalizing Problem Behaviors

In the literature, two conceptions of moral competence can be distinguished: the social-cognitive framework and the virtue approach. Within the social-cognitive framework, moral maturity is regarded as “the capacity to make decisions and judgments which are moral (that is, based on internal principles) and to act in accordance with such judgments” (Kohlberg, 1964, p. 425). According to this view, moral competence is conceived as moral judgment ability which develops with age along a six-stage process involving the transforming egocentric thinking to more social and ethical oriented thinking (Kohlberg, 1984; Gibbs et al., 2007; Gibbs, 2014). Recently, the last two and less common “principled” stages were reinterpreted as representing reflective philosophies indicative of an “existential developmental phase” (Gibbs et al., 2007; Gibbs, 2014).

Alternatively, using a virtue approach, Park and Peterson (2006) stated that “moral competence among the adolescents can be approached in terms of good character” (p. 891) where good character is “a family of positive traits reflected in thoughts, feelings and behavior” (p. 893). In their Values in Action (VIA) Classification framework, 24 character strengths, such as “appreciation of beauty and excellence” and “self-regulation,” are proposed (Park and Peterson, 2006). Moral competence as virtues is also emphasized in the management field. For example, Morales-Sánchez and Cabello-Medina (2013) proposed that “moral virtues in the workplace would be moral competencies” (p. 721). Martin and Austin (2010) proposed that moral competences consist of ten positive traits, including keeping promises and active caring about others.

These two conceptions also differ in their measurement of moral competence. Compared with moral judgment which is commonly assessed by dilemma-based moral judgment interviews or dilemma-free production tests (Gibbs et al., 2007), moral virtues are usually measured by self-report methods (e.g., Seider et al., 2013; Wang et al., 2015). Besides, scholars have developed validated self-report measures of moral competence as virtues in both the Western (Park and Peterson, 2006) and Chinese (Shek et al., 2007) contexts.

Despite these differences, the two approaches share a common feature regarding the relationship between moral competence and adolescent development. It is widely acknowledged that developmentally delayed moral judgment is associated with adolescent immoral behavior, such as delinquency (Kohlberg, 1984; Stams et al., 2006; Gibbs, 2014). Within the virtue approach, it is asserted that a good life can be constructed by building and promoting one’s strength of character (Seligman, 2002). Likewise, in their review of successful PYD programs in the United States, Catalano et al. (2002) asserted that moral competence is an important PYD construct which positively influences youth developmental outcomes. Research showed that certain character traits are negatively related to youth problems (e.g., bullying) while positively related to desired achievement and wellness (e.g., happiness) (Park, 2004; de Kemp et al., 2009). For instance, Kim and Brody (2005) reported that adolescent self-regulation negatively predicted the externalizing (i.e., delinquency) and internalizing problems (e.g., depression).

However, the related empirical evidence is far from conclusive. First, instead of examining how moral virtues affect delinquency, the prior studies primarily focused on the nature of virtues or virtue development (Berkowitz and Hoppe, 2009; Wang et al., 2015). Second, among the limited studies, the findings were not conclusive. For example, in Seider et al.’s (2013) study, although students’ misconduct in the school was negatively correlated with their integrity and perseverance, the predicting effect of the two attributes was not identified in the regression analyses. Third, the inconclusiveness of the picture may also be, at least in part, due to the recruitment of small samples and the lack of longitudinal studies (Seider et al., 2013).

Another unresolved theoretical issue is whether and how early development of moral virtues affects the developmental trajectories of problem behaviors. It is commonly conjectured that better development of positive characters including moral competence helps adolescents to cope with life stress more constructively, thus establishing a solid foundation for adolescent long-term adaptive adjustment (Lerner et al., 2013; Shek and Leung, 2013). For example, in Shek and Yu’s (2012) study, the adolescents who joined a PYD program demonstrated a slower increment of delinquency compared with their peers who did not receive the program. Two recent studies conducted by Shek and Lin (2016a,b) directly related the developmental trajectory of delinquency to a composite measure of adolescent PYD (e.g., emotional competence, moral competence and spirituality). Discordant with common sense, it was found that better initial PYD status was associated with a faster increasing rate of delinquency over the adolescent years. The researchers suggested that the experimentation of “safer” delinquent behaviors or other problem acts among the better-functioning adolescents may help explain the unexpected findings (Shek and Lin, 2016a). However, how the developmental trajectory of externalizing problem behaviors is affected by the level of moral competence remains unknown given such limited evidence.

To fill these research gaps, using a longitudinal design, this study investigated how moral competence defined by the virtues predict the level of as well the rate of change in externalizing problems among adolescents with specific reference to the Chinese context. As such, this study focused on three virtues that have traditionally been emphasized in the Chinese culture and echo the Western conception of character strengths.

The first virtue is the Confucian emphasis of “a perfect and virtuous man” (“Junzi” in Chinese) that connotes high moral expectation for oneself (Chan, 2008). It is strongly emphasized that one should strive to have high morality in Chinese education (Wang, 2004; Gu, 2006). This virtue is conceptually consistent with such virtues as “appreciation of beauty and excellence” and “courage” in the VIA model (Park and Peterson, 2006). The second virtue is “Xin” (trustworthiness), which is one of the five cardinal virtues of Confucianism (Shek et al., 2013). This virtue echoes the character strength of “persistence in one’s words and behaviors” in the VIA model (Park and Peterson, 2006) which has been emphasized by other researchers (Jormsri et al., 2005; Schniter et al., 2013). The third one is self-reflection and self-evaluation. The Confucians always advocate continuous and frequent self-evaluation and reflection as a way to examine one’s own morality (Wang, 2004). This attribute is also included in the VIA model (Park and Peterson, 2006) and is subsumed under “prudence” in the moral competency model proposed by Morales-Sánchez and Cabello-Medina (2013).

Another operational question related to conducting longitudinal study on problem behaviors is the assessment issue. Under the social-cognitive framework, some studies compared the officially defined delinquents with the non-delinquent controls and identified a clear negative linkage between moral judgment and delinquency (See Stams et al., 2006 for a review). In contrast, the relationships between moral judgment and self-report delinquency were much weaker (e.g., Tarry and Emler, 2007; Beerthuizen et al., 2013). While the known-group comparison involving official records is more objective, it has well-documented shortcomings such as its vulnerability to social class and race biases (Emler and Tarry, 2007). Hence, many researchers used the self-report methods (e.g., Emler and Tarry, 2007). Moreover, it is difficult, if not impossible, to use the officially defined delinquent behavior in longitudinal studies. This may be one of the reasons that almost all studies adopting this method are cross-sectional (Stams et al., 2006). Thus, similar to other longitudinal research (e.g., Raaijmakers et al., 2005), the present study employed self-report externalizing problem behaviors.

### Spirituality and Externalizing Problem Behaviors

Although there is no agreement on the definition of spirituality, some essential elements are generally highlighted, including the purpose and meaning of life, hope, compassion, relationship, connectedness and beliefs in the higher being (Shek and Ho, 2016). As King and Boyatzis (2004) asserted, adolescence is “an age period of intense ideological hunger, a striving for meaning and purpose, and desire for relationships and connectedness” (p. 2). This notion is clearly supported by the human history and research studies (Shek, 2012). For example, Astin et al. (2005) reported that approximately 80% of the freshmen in the United States regarded themselves as “spiritual beings” who were interested in spirituality and believed in the sacredness of life. Similar findings were mentioned in other studies (Gallup and Bezilla, 1992; Bibby, 2006).

Many theorists contended that spirituality is an important source of resilience, which is an asset in coping with negative life experiences and can help adolescents deal with life difficulties and problems in an adaptive way (Crawford et al., 2006; Koenig, 2010). This argument echoes the theory proposed by Frankl (1967) which states that psychological and adjustment problems will fill the existential vacuum caused by the loss of meaning of life. Empirically, higher levels of spirituality are closely related to higher life satisfaction, less depressive symptoms, better self-images and less problem behaviors among adolescents (e.g., Wong et al., 2006; Kub and Solari-Twadell, 2013; Salas-Wright et al., 2013).

Despite the general proposal that spirituality is negatively related to adolescent problem behavior, there are still several research gaps in the literature. First, most studies conducted in the Western communities adopted the concept of religiosity without differentiating between the concepts of spirituality and religiosity, leading to a concept called “religiosity/spirituality (R/S)” (Kub and Solari-Twadell, 2013). Although it makes sense in the contexts in which the definition of spirituality is broad enough to include religious spirituality as an essential element (Worthington et al., 2011), the situation may be different in the Chinese communities where religion is not an indispensable part of many people’s lives. For example, a recent study reported that nearly 70% of the Chinese-speaking adolescents in Hong Kong did not have a religious belief and religiosity only accounted for a very small part of their life satisfaction (Yuen et al., 2016). Thus, for the Chinese adolescents, religion may not play a major role in the scope of spirituality compared with other critical components such as “the meaning of life” (Lau, 2006). This may be the reason why the meaning or purpose of life is usually the focus of the studies and measures pertaining to spirituality of the Chinese adolescents (Shek et al., 2007; Shek, 2010, 2012).

Second, similar to the case of moral competence, there is a lack of longitudinal research on the relationship between spirituality and adolescent problem behaviors regarding the level and the rate of change over time. To fill these two major knowledge gaps, the present study investigated the predictive effects of Chinese adolescents’ spirituality on both the levels and the developmental trajectories of risk and delinquent behavior as well as intentions to engage in problem behaviors.

### The Present Study

The present study attempted to investigate whether adolescents’ moral competence and spirituality predicted the levels and change courses of the externalizing problem behaviors indexed by risk and delinquent behavior and problem behavioral intention across the high school years (i.e., from Grade 7 to Grade 12). There are several research questions in this study. First, we asked what the developmental trajectories of problem behaviors over the high school years are. Based on the previous findings (Shek and Lin, 2016a,b), we expected that there would be an increasing trend in adolescent risk and delinquent behavior (Hypothesis 1a) and problem behavioral intention (Hypothesis 1b) over time.

Second, we asked whether the moral competence and spirituality predicted the levels of risk and delinquent behavior and problem behavioral intention. Based on the PYD perspective and previous findings (Kim and Brody, 2005; Salas-Wright et al., 2013), we expected that adolescents with lower levels of moral competence or spirituality would demonstrate higher levels of risk and delinquent behavior (Hypothesis 2a and Hypothesis 2b, respectively) and higher problem behavioral intention (Hypothesis 2c and Hypothesis 2d, respectively).

Third, we asked whether moral competence and spirituality predicted the change rates of risk and delinquent behavior and problem behavioral intention. Based on the previous findings, we expected that lower levels of moral competence or spirituality would predict faster linear growth rates of risk and delinquent behavior (Hypothesis 3a and Hypothesis 3b, respectively) and problem behavioral intention (Hypothesis 3c and Hypothesis 3d, respectively).

Regarding the predictive effects of moral competence and spirituality on the quadratic changes of two problem behavior indicators, we did not make any specific hypotheses due to the lack of scientific literature on the theorization. As the previous studies have provided support for the influence of the socio-demographic factors (e.g., gender and economic disadvantage) on the problem behaviors (Shek and Lin, 2016b), the present study considered three socio-demographic factors as control variables.

## Materials and Methods

### Participants and Procedures

The present study used data obtained in a large-scale longitudinal project in Hong Kong, which commenced in 2009–2010 school year. In this project, a cohort of Grade 7 students from 28 randomly selected secondary schools in Hong Kong was followed through in six consecutive years. These students completed the same battery of measures each year, resulting in six waves of data. The interval between the first five successive waves was approximately 1 year while Wave 6 data collection took place approximately 10 months after Wave 5. This is because Grade 12 students in Hong Kong need to sit the public examination in the last few months during their high school study.

The sample consisted of 3,328 Grade 7 students (*M*_age_ = 12.59 ± .74 years) at Wave 1, including 1,735 (52.1%) boys, 1,584 (47.6%) girls and 9 (0.3%) who did not provide gender information. According to Shek et al. (2014), these participants constitute a representative sample of adolescents in Hong Kong as they compared favorably with general secondary school student population in Hong Kong regarding demographic characteristics. From Wave 2 to Wave 6, the number of participants joining the project decreased from 2,905 to 2,385. Therefore, the present attrition rates ranged between 12.7% and 28.3% across waves, which were favorable compared to previous longitudinal research lasting for multiple years (Bongers et al., 2003).

Before Wave 1 data collection, the participating schools and students’ parents were well informed that the data collected from the students would be kept confidential and used only for research purposes. Their written consent was obtained. Before each wave of data collection, the principles of voluntary participation and confidentiality were explained to the participating students and their written consent was also obtained. In each occasion of data collection, the questionnaires were administrated by the trained research staff in quiet classrooms in each participating school. The participants were instructed to give their own true opinion for each question and not to discuss with their classmates.

### Instruments

The project included multiple measures, including the demographic characteristics (e.g., gender), positive developmental assets (e.g., moral competence), problem behaviors and family functioning (see Shek et al., 2014). The positive developmental assets were assessed using the short version of the Chinese Positive Youth Development Scale (CPYDS), which was developed and validated by Shek et al. (2007) and Shek and Ma (2010). Noteworthy, the CPYDS has been widely adopted to assess PYD attributes among Chinese adolescents (e.g., Shek and Lin, 2016a; Shek et al., 2016). A short version was used to reduce fatigue concerning the length of the whole questionnaire. To ensure the reliability and validity, three items having the highest factor loadings within each subscale were selected to form the short version of the CPYDS (Shek and Ma, 2010). The present study focused on four measures: moral competence, spirituality, delinquency and the problem behavioral intention.

#### Moral Competence

Moral competence was assessed by three items: (1) “I have high moral expectation about my behavior”; (2) “I will fulfill my promise”; and (3) “I have the habit of self-evaluation.” As aforementioned, these items are closely related to moral virtues in the Chinese Confucian thoughts. Moreover, they echo the character strengths in the VIA model: “appreciation for excellence,” “keeping one’s promise” and “self-reflection” respectively (Park and Peterson, 2006). A 6-point rating scale (1 = strongly disagree, 6 = strongly agree) was used and moral competence was computed as the average score across the three items. In the current study, reliability of the 3-item scale was supported (see **Table 1**).

**Table 1:** Descriptive statistics of key variables and reliability (Cronbach’s α) of scales across six waves.

Variables	Wave 1		Wave 2		Wave 3		Wave 4		Wave 5		Wave 6	
						
	Mean (*SD*)	Reliability	Mean (*SD*)	Reliability	Mean (SD)	Reliability	Mean (*SD*)	Reliability	Mean (*SD*)	Reliability	Mean (*SD*)	Reliability
Moral competence	4.37 (0.91)	0.73	4.41 (0.85)	0.75	4.46 (0.81)	0.74	4.49 (0.80)	0.75	4.50 (0.75)	0.70	4.55 (0.74)	0.70
Spirituality	5.14 (1.32)	0.88	5.01 (1.29)	0.89	5.01 (1.26)	0.90	4.96 (1.23)	0.91	4.92 (1.24)	0.92	4.95 (1.23)	0.92
Risk and delinquent behavior	0.39 (0.47)	0.70	0.46 (0.54)	0.73	0.45 (0.50)	0.67	0.47 (0.49)	0.64	0.51 (0.56)	0.71	0.49 (0.55)	0.69
Behavioral intention	1.26 (0.39)	0.64	1.34 (0.45)	0.68	1.37 (0.46)	0.69	1.42 (0.47)	0.66	1.49 (0.50)	0.66	1.58 (0.50)	0.65


#### Spirituality

The spirituality subscale of the CPYDS was modeled after the Chinese version of the Purpose in Life Questionnaire (Shek et al., 2007). The three items employed in the present study assessed whether the respondent perceived the life as colorful, meaningful and purposeful (e.g., “My life is dull versus colorful” and “My life is empty versus full”). The previous studies showed that the scale had good psychometric properties (Shek and Ma, 2010). The average score across the three items was calculated and a higher score represents a higher level of spirituality. As shown in **Table 1**, the 3-item spirituality scale showed good reliability in the current study.

#### Risk and Delinquent Behavior

This construct was assessed by the frequency of having 12 problem behaviors including those are illegal and acts are not so serious but considered as risk in the previous 1 year. These behaviors included “stealing,” “cheating,” “truancy,” “running away from home,” “damaging others’ properties,” “beating others,” “having sexual intercourse with others,” “gang fighting,” “speaking foul language,” “staying outside the home overnight without parental consent,” “bullying” and “trespassing.” A 7-point scale was used with a higher score representing a higher level of risk and delinquent behavior. A composite score was calculated by averaging the scores across all items and the scale reliability was supported (see **Table 1**).

#### Problem Behavioral Intention

On the 4-point scale (1 = absolutely will not, 4 = absolutely will), the participants reported their intention to engage in five problem behaviors, including drinking, smoking, gambling, drug abuse and having sexual intercourse, in the next 2 years. An average score of the scale was calculated for each participant. The 5-item scale demonstrated acceptable reliability (see **Table 1**).

#### Control Variables

Three socio-demographic factors were considered as control variables, including gender, family economic disadvantage and family intactness. In Hong Kong, living on welfare of “Comprehensive Social Security Assistance Scheme” (CSSA) provided by the government can be regarded as a valid index of family economic disadvantage. In the current study, 225 (6.76%) participants living on CSSA in Wave 1 were defined as having family economic disadvantage. Other 2,606 (78.31%) adolescents not living on CSSA were regarded as not having economic disadvantage. Family intactness was operationalized as the marital status of the participants’ parents. A total of 2,781 (83.56%) participants who indicated that their parents were in the first marriage were classified as the intact family group while 515 (15.47%) participants whose parents were divorced, separated or in their second marriage were categorized into non-intact family group. The participants with missing values in the control variables were excluded from statistical analysis involving the control variables.

### Attrition Analyses and Missing Values Imputation

We compared the participants who had been joining the study with those who dropped out for each wave in terms of their baseline situation at Wave 1. Compared with the dropouts, those adolescents participating in the study were slightly younger, consisted of a higher proportion of girls, had higher levels of moral competence and spirituality and had lower levels of externalizing problem behaviors. To reduce bias induced by the systematic attrition, we used multiple imputation to impute the missing values of the core research variables. Following the guidelines given by Asendorpf et al. (2014), we applied the “multiple imputation” procedure of SPSS, applied the option of “Predictive Mean Matching” (PMM) and changed the default five imputations to forty. Besides, the three control variables were included as auxiliary variables in addition to the core research variables in the imputation equation. After this procedure, we had forty imputed datasets in addition to the original one.

After multiple imputation, statistical analyses including correlation analyses and individual growth curve (IGC) analyses were performed based on the original data set and the forty imputed data sets separately. For each statistical analysis parameter, an average value calculated across the imputation samples was the pooled result (Rubin, 1987). Analyses based on the original data set and the imputed data sets yielded similar results, suggesting that attrition was not a big problem in the present study. In the sections below, the pooled results were reported for correlations and the IGC models. This approach has been widely used recently and is highly recommended to minimize the attrition effect in the longitudinal studies (e.g., Asendorpf et al., 2014; Huijbers et al., 2015).

### Data Analysis

Data analysis was conducted using SPSS for Windows, version 23.0 (IBM Corp., Somers, NY, United States). The reliability analysis and descriptive analyses were first conducted, followed by the correlation analyses and IGC analyses. The IGC analyses were conducted to examine the developmental trajectories of the adolescent problem behaviors and whether they were predicted by moral competence and spirituality, with the effects of the socio-demographic variables being controlled.

Individual growth curve is an analytic approach that has been frequently used to track the adolescent developmental trajectory and explore the individual differences in the growth rate (Shek and Yu, 2012; Shek and Lin, 2016b). Following the analysis procedures suggested by Shek and Ma (2011), we utilized a 2-level hierarchical model, which nested time (level 1) into individual (level 2). The model was tested in two steps. First, for each problem behavior indicator, three unconditional models using level-1 predictors (Model 1: unconditional mean model; Model 2: linear growth model; Model 3: quadratic growth model) were examined to identify the growth curve. Time was coded as 0, 1, 2, 3, 4, and 4.83 for Wave 1, Wave 2, Wave 3, Wave 4, Wave 5 and Wave 6, respectively. Second, three control variables and the two predictor variables were examined as time-invariant covariates to explore any individual variability in the shape of the growth trajectories with regard to the two problem behaviors. To illustrate, the 2-level model is depicted as follows:

$$Level-1:Y_{ij}=\beta_{0j}+\beta_{1j}\left(Time\right)+\beta_{2j}\left({Time}^{2}\right)+r_{ij}$$

$$\begin{array}{ccc} \text{Level - 2: }\beta_{0j} & = & \gamma_{00}+\gamma_{01}(\text{gender})+\\ & & \gamma_{02}(\text{economicdisadvantage})+\\ & & \gamma_{03}(\text{familyintactness})+\\ & & \gamma_{04}(\text{moralcompetence})+\\ & & \gamma_{05}(\text{spirituality})+u_{0j}\\ \beta_{1j} & = & \gamma_{10}+\gamma_{11}(\text{gender})+\\ & & \gamma_{12}(\text{economicdisadvantage})+\\ & & \gamma_{13}(\text{familyintactness})+\\ & & \gamma_{14}(\text{moralcompetence})+\\ & & \gamma_{15}(\text{spirituality})+u_{1j}\\ \beta_{2j} & = & \gamma_{20}+\gamma_{21}(\text{gender})+\\ & & \gamma_{22}(\text{economicdisadvantage})+\\ & & \gamma_{23}(\text{familyintactness})+\\ & & \gamma_{24}(\text{moralcompetence})+\\ & & \gamma_{25}(\text{spirituality})+u_{2j} \end{array}$$

In each analysis, the model fit was indexed by three indices: -2log likelihood (i.e., deviance), Akaike Information Criterion (AIC) and Bayesian Information Criterion (BIC), with a small index indicating a better model fit (Shek and Ma, 2011). Furthermore, the individual growth curves were plotted using the prototypical values.

Prior to the IGC analyses, the three control variables were dummy coded as follows: female = –1, male = 1; having economic disadvantage = -1, not having economic disadvantage = 1; non-intact family = -1, intact family = 1. Besides, the two predictors (i.e., moral competence and spirituality) were standardized so that the gamma coefficients of each predictor in intercept, linear slope and quadratic slope represented the changes of problem behavior under investigation, in mean value, rate of linear change and curvature, respectively, per unit of change in the predictor. Furthermore, a natural logarithmic transformation was performed for each problem behavior indicator to reduce the skewness in data distribution.

## Results

The percentages of the adolescents who committed risk and delinquent behaviors at least once in the past 1 year are demonstrated in **Table 2**. Overall speaking, cheating and speaking foul language were the two most common misconduct. Specifically, the percentages of the adolescents who had ever cheated and spoken foul language more than once in the past 1 year ranged between 56.61 and 62.66%, and between 68.35 and 70.90%, respectively, across the six waves. Further examination of the inter-item correlations regarding delinquency showed acceptable mean inter-item correlations (i.e., around.30) across the six waves (Schmitt, 1996). Similar results were observed regarding the items in the problem behavioral intention measure.

**Table 2:** Percentages of participants committing each risk or delinquent behavior at least once in the past one year in each wave.

Risk or delinquent behaviors		Wave 1	Wave 2	Wave 3	Wave 4	Wave 5	Wave 6
1	Stealing	10.07	9.48	7.45	6.08	5.69	5.03
2	Cheating	60.76	62.66	60.29	60.16	62.36	56.61
3	Truancy	3.29	4.07	4.42	4.56	6.15	6.07
4	Running away from home	3.98	4.15	4.06	2.63	2.60	2.08
5	Damaging others’ properties	13.45	12.45	8.62	8.06	6.82	5.59
6	Beating others	11.71	10.18	7.32	5.19	4.69	4.77
7	Having sexual intercourse with others	0.66	1.14	1.05	1.47	2.51	2.65
8	Gang fighting	3.34	3.36	2.26	1.98	2.14	2.13
9	Speaking foul language	69.32	70.90	68.85	69.17	70.49	68.35
10	Staying outside the home overnight without parental consent	3.02	3.49	3.45	3.88	4.44	5.05
11	Bullying or threatening others	15.65	14.57	10.17	9.09	7.62	6.29
12	Trespassing	3.77	3.18	2.72	2.56	2.22	2.08


As shown in **Table 3**, moral competence and spirituality levels were associated with risk and delinquent behavior and problem behavioral intention levels across the six waves. The IGC analyses were then conducted and the results are illustrated in **Tables 4**–**7**.

**Table 3:** Pooled correlations among variables based on forty computed data sets.

Variables		1	2	3	4	5	6	7	8	9	10	11	12	13	14	15	16
1	W1 RDE	-															
2	W2 RDE	0.61	-														
3	W3 RDE	0.54	0.65	-													
4	W4 RDE	0.47	0.57	0.65	-												
5	W5 RDE	0.46	0.55	0.61	0.69	-											
6	W6 RDE	0.44	0.51	0.56	0.62	0.69	-										
7	W1 Behavioral intention	0.49	0.40	0.35	0.30	0.30	0.29	-									
8	W2 Behavioral intention	0.41	0.52	0.41	0.35	0.36	0.33	0.56	-								
9	W3 Behavioral intention	0.40	0.45	0.54	0.42	0.41	0.38	0.48	0.61	-							
10	W4 Behavioral intention	0.35	0.43	0.45	0.52	0.46	0.43	0.44	0.57	0.65	-						
11	W5 Behavioral intention	0.33	0.39	0.42	0.44	0.54	0.45	0.42	0.53	0.59	0.69	-					
12	W6 Behavioral intention	0.33	0.39	0.39	0.42	0.46	0.53	0.38	0.49	0.54	0.63	0.70	-				
13	Gender^a^	0.08	0.09	0.12	0.16	0.20	0.17	0.07	0.05	0.09	0.12	0.16	0.15	-			
14	Economic disadvantage^b^	-0.02	-0.06	-0.05	-0.05	-0.03	-0.04	0.02	-0.03	-0.04	-0.01	-0.02	-0.01	0.03	-		
15	Family intactness^c^	-0.09	-0.10	-0.09	-0.07	-0.06	-0.06	-0.08	-0.08	-0.09	-0.06	-0.05	-0.07	0.005	0.35	-	
16	Moral competence	-0.32	-0.28	-0.25	-0.21	-0.22	-0.20	-0.21	-0.20	-0.22	-0.21	-0.19	-0.17	-0.12	0.02	0.08	-
17	Spirituality	-0.27	-0.21	-0.18	-0.14	-0.14	-0.13	-0.22	-0.16	-0.17	-0.13	-0.14	-0.11	-0.05	0.06	0.10	0.40



*Correlation coefficients with an absolute value greater than 0.04 are significant at *p* < 0.05. ^*a*^Male = 1, Female = -1; ^*b*^Having economic disadvantage = -1, Not having economic disadvantage = 1; ^*c*^Non-intact = -1, Intact = 1. W1, Wave 1; W2, Wave 2; W3, Wave 3; W4, Wave 4; W5, Wave 5; W6, Wave 6; RDE, risk and delinquent behavior.*

**Table 4:** Pooled results of IGC models with level-1 predictors for adolescent risk and delinquent behavior.

		Model 1		Model 2		Model 3	
			
		Estimate	*SE*	Estimate	*SE*	Estimate	*SE*
*Fixed effects*							
**Intercept**	β_0*j*_						
Intercept	γ_00_	0.343***	0.0043	0.305***	0.0046	0.296***	0.0048
**Linear slope**	β_1*j*_						
Time	γ_10_			0.015***	0.0011	0.030***	0.0032
**Quadratic slope**	β_2*j*_						
Time^2^	γ_20_					-0.003***	0.0006
*Random effects*							
Level 1 (within)							
Residual	*r_ij_*	0.0414***	0.0005	0.0340***	0.0004	0.0315***	0.0004
Level 2 (between)							
Intercept	*u*_0*j*_	0.0548***	0.0015	0.0534***	0.0018	0.0513***	0.0019
Time	*u*_1*j*_			0.0020***	0.0001	0.0092***	0.0009
Time^2^	*u*_2*j*_					0.0003***	0.00003
*Fit statistics*							
Deviance		390.13		-750.27		-925.03	
AIC		396.13		-738.27		-905.03	
BIC		419.82		-690.86		-826.02	
df		3		6		10	


*Adolescent risk and delinquent behavior is log-transformed using natural logarithm. Model 1, unconditional mean model; Model 2, unconditional linear growth model; Model 3, unconditional quadratic growth model. AIC, Akaike Information Criterion; BIC, Bayesian Information Criterion. ^∗∗∗^*p* < 0.001.*

**Table 5:** Pooled results of IGC models with level-2 predictors for adolescent risk and delinquent behavior.

		Model 4		Model 5	
			
		Estimate	*SE*	Estimate	*SE*
*Fixed effects*					
**Intercept**	β_0*j*_				
Intercept	γ_00_	0.325***	0.0101	0.315***	0.0094
Gender^a^	γ_01_	0.017**	0.0052	0.006	0.0049
Economic disadvantage^b^	γ_02_	-0.002	0.0104	0.0002	0.0097
Family intactness^c^	γ_03_	-0.033***	0.0079	-0.021**	0.0073
Moral competence	γ_04_			-0.072***	0.0053
Spirituality	γ_05_			-0.050***	0.0053
**Linear slope**	β_1*j*_				
Intercept	γ_10_	0.043***	0.0068	0.044**	0.0068
Gender^a^	γ_11_	0.014***	0.0035	0.016***	0.0035
Economic disadvantage^b^	γ_12_	-0.015*	0.0070	-0.015*	0.0070
Family intactness^c^	γ_13_	0.003	0.0053	0.0005	0.0053
Moral competence	γ_14_			0.007	0.0039
Spirituality	γ_15_			0.013**	0.0038
**Quadratic slope**	β_2*j*_				
Intercept	γ_20_	-0.005***	0.0013	-0.006***	0.0013
Gender^a^	γ_21_	-0.001	0.0007	-0.001	0.0007
Economic disadvantage^b^	γ_22_	0.003	0.0014	0.003	0.0014
Family intactness^c^	γ_23_	-0.0001	0.0010	0.0001	0.0010
Moral competence	γ_24_			-0.0004	0.0008
Spirituality	γ_25_			-0.001	0.0007
*Random effects*					
Level 1 (within)					
Residual	*r_ij_*	0.0313***	0.0005	0.0313***	0.0005
Level 2 (between)					
Intercept	*u*_0*j*_	0.0495***	0.0021	0.0393***	0.0018
Time	*u*_1*j*_	0.0095***	0.0010	0.0092***	0.0010
Time^2^	*u*_2*j*_	0.0003***	0.00004	0.0003***	0.00004
*Fit statistics*					
Deviance		-984.63		-1412.07	
AIC		-946.63		-1362.07	
BIC		-799.89		-1168.99	
df		19		25	


*Adolescent risk and delinquent behavior is log-transformed using natural logarithm. Model 4, conditional growth curve model (only with socio-demographic variables); Model 5, conditional growth curve model (adding moral competence). ^*a*^Male = 1, Female = -1; ^*b*^Having economic disadvantage = -1, Not having economic disadvantage = 1; ^*c*^Non-intact = -1, Intact = 1. AIC, Akaike Information Criterion; BIC, Bayesian Information Criterion. ^∗^*p* < 0.05. ^∗∗^*p* < 0.01. ^∗∗∗^*p* < 0.001.*

**Table 6:** Pooled results of IGC models with level-1 predictors for adolescent problem behavioral intention.

		Model 1		Model 2		Model 3	
				
		Estimate	*SE*	Estimate	*SE*	Estimate	*SE*
*Fixed effects*							
**Intercept**	β_0*j*_						
Intercept	γ_00_	0.308***	0.0041	0.194***	0.0043	0.205***	0.0044
**Linear Slope**	β_1*j*_						
Time	γ_10_			0.046***	0.0011	0.029***	0.0032
**Quadratic Slope**	β_2*j*_						
Time^2^	γ_20_					0.003***	0.0006
*Random effects*							
Level 1 (within)							
Residual	*r_ij_*	0.0468***	0.0005	0.0328***	0.0004	0.0303***	0.0004
Level 2 (between)							
Intercept	*u*_0*j*_	0.0481***	0.0013	0.0441***	0.0015	0.0385***	0.0016
Time	*u*_1*j*_			0.0020***	0.0001	0.0100***	0.0010
Time^2^	*u*_2*j*_					0.0003***	0.00003
*Fit statistics*							
Deviance		2068.82		-1674.40		-1966.96	
AIC		2074.82		-1662.40		-1946.96	
BIC		2098.53		-1614.99		-1867.95	
df		3		6		10	


*Adolescent problem behavioral intention is log-transformed using natural logarithm. Model 1, unconditional mean model; Model 2, unconditional linear growth model; Model 3, unconditional quadratic growth model. AIC = Akaike Information Criterion; BIC = Bayesian Information Criterion. ^∗∗∗^*p* < 0.001*

**Table 7:** Pooled results of IGC models with level-2 predictors for adolescent problem behavioral intention.

		Model 4		Model 5	
			
		Estimate	*SE*	Estimate	*SE*
*Fixed effects*					
**Intercept**	β_0*j*_				
Intercept	γ_00_	0.212***	0.0091	0.206***	0.0088
Gender^a^	γ_01_	0.012*	0.0047	0.005	0.0046
Economic disadvantage^b^	γ_02_	0.021*	0.0095	0.023*	0.0091
Family intactness^c^	γ_03_	-0.037***	0.0071	-0.029***	0.0069
Moral competence	γ_04_			-0.038***	0.0050
Spirituality	γ_05_			-0.040***	0.0050
**Linear slope**	β_1*j*_				
Intercept	γ_10_	0.041***	0.0067	0.041***	0.0067
Gender^a^	γ_11_	0.009*	0.0035	0.008*	0.0035
Economic disadvantage^b^	γ_12_	-0.020**	0.0070	-0.020**	0.0070
Family intactness^c^	γ_13_	0.008	0.0052	0.008	0.0053
Moral competence	γ_14_			-0.010*	0.0038
Spirituality	γ_15_			0.007	0.0038
**Quadratic slope**	β_2*j*_				
Intercept	γ_20_	0.002	0.0013	0.002	0.0013
Gender^a^	γ_21_	-0.0002	0.0007	0.0001	0.0007
Economic disadvantage^b^	γ_22_	0.003*	0.0013	0.003*	0.0013
Family intactness^c^	γ_23_	-0.0013	0.0010	-0.001	0.0010
Moral competence	γ_24_			0.002*	0.0007
Spirituality	γ_25_			-0.001	0.0007
*Random effects*					
Level 1 (within)					
Residual	*r_ij_*	0.0299***	0.0005	0.0299***	0.0005
Level 2 (between)					
Intercept	*u*_0*j*_	0.0372***	0.0017	0.0330***	0.0016
Time	*u*_1*j*_	0.0099***	0.0010	0.0098***	0.0010
Time^2^	*u*_2*j*_	0.0003***	0.00004	0.0003***	0.00004
*Fit statistics*					
Deviance		-1932.51		-2178.10	
AIC		-1894.51		-2128.10	
BIC		-1747.77		-1935.02	
df		19		25	


*Adolescent problem behavioral intention is log-transformed using natural logarithm. Model 4, conditional growth curve model (only with socio-demographic variables); Model 5, conditional growth curve model (adding moral competence). ^*a*^Male = 1, Female = -1; ^*b*^Having economic disadvantage = -1, Not having economic disadvantage = 1; ^*c*^Non-intact = -1, Intact = 1. AIC, Akaike Information Criterion; BIC, Bayesian Information Criterion. ^∗^*p* < 0.05. ^∗∗^*p* < 0.01. ^∗∗∗^*p* < 0.001.*

### Risk and Delinquent Behavior Over Time and Its Predictors

**FIGURE 1:**
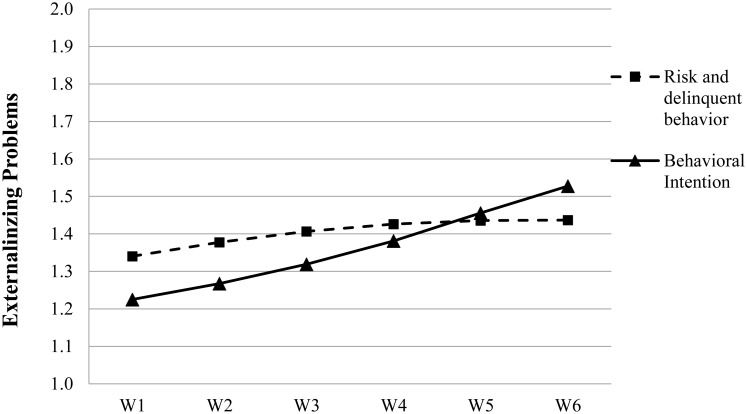
Growth trajectory of adolescent risk and delinquent behavior and problem behavioral intention for the overall sample based on Model 3 in **Tables 4**, **6**, respectively.

The results of the unconditional mean model (Model 1 in **Table 4**) indicated an intra-class correlation coefficient (ICC) of.569, which implied that 56.9% of the total variance in adolescent risk and delinquent behavior was related to the inter-individual differences. Thus, there is necessity to perform the multi-level analyses (Shek and Ma, 2011). Moreover, the unconditional quadratic model (i.e., Model 3) showed a better model fit than the unconditional linear model, i.e., Model 2 [Δχ^2^_(4)_ = 174.76; *p* < 0.001; ΔAIC = 166.76; ΔBIC = 135.16]. According to Model 3 (see **Table 4**), there was a significant linear increase in the whole sample’s average level of risk and delinquent behavior (β = 0.030, *p* < 0.001). Hence, Hypothesis 1a was supported. A negative and significant quadratic effect was also observed (β = –0.003, *p* < 0.001), although the quadratic change (-0.003) was small compared to the linear increasing trend (0.030). These results suggested that adolescent risk and delinquent behavior increased during the secondary school years, but such an increasing trend slightly slowed down over time (see **Figure 1**).

As the variability was significant in the intercept, linear slope and quadratic slope in Model 3, we examined the predictive effects in the intercept, linear and quadratic parameters. Model 4 only considered the control variables while Model 5 considered all predictors including moral competence and spirituality. As shown in **Table 5**, compared to Model 4, Model 5 had a better model fit [Δχ^2^_(6)_ = 427.44; *p* < 0.001; ΔAIC = 415.44; ΔBIC = 369.10]. Also, Model 5 better fitted the data than did Model 3 [Δχ^2^_(15)_ = 487.04; *p* < 0.001; ΔAIC = 457.04; ΔBIC = 342.97]. Therefore, the results were interpreted based on Model 5.

**FIGURE 2:**
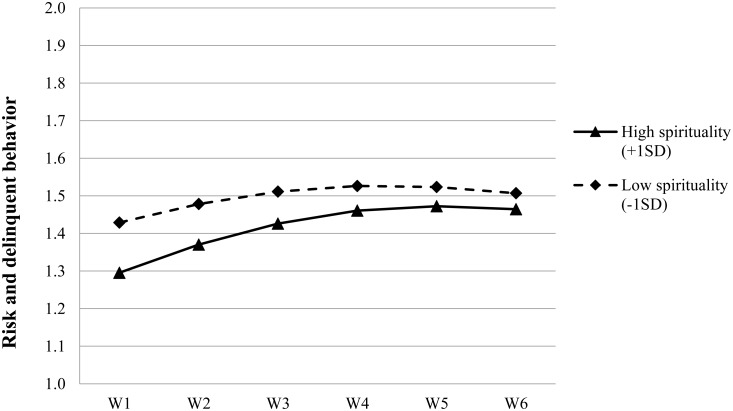
Growth trajectories of adolescent risk and delinquent behavior as a function of spirituality. The figures were plotted based on Model 5 in **Table 5**. High level indicates 1 SD higher than the mean value; low level indicates 1 SD lower than the mean value.

As shown in Model 5, adolescent risk and delinquent behavior increased over time (β = 0.044, *p* < 0.001) with the growth rate slightly slowing down (β = –0.006, *p* < 0.001). For the intercept, family intactness, moral competence and spirituality were the significant predictors of the variability. The results indicated that the students living in non-intact families reported a higher level of risk and delinquent behavior than those living in intact families. *Post hoc* analyses suggested that such a difference remained at a significant level across the six waves (*t*s > 3.29, *p*s < 0.001). Furthermore, the adolescents with a lower level of moral competence or spirituality reported a higher level of risk and delinquent behavior at the initial stage, giving support for Hypotheses 2a and 2b. As for the linear change rate, gender, economic disadvantage and spirituality were the significant predictors while moral competence was not (i.e., Hypothesis 3a was not supported). Specifically, the growth rate of risk and delinquent behavior was slower among the female adolescents, the adolescents without family economic disadvantage and the adolescents with a lower level of spirituality (see **Figure 2**). The predicting effect of spirituality on the linear growth rate of risk and delinquent behavior was just contrary to our Hypothesis 3b.

It is noteworthy that the greater increasing rate as suggested by the gamma coefficients in the adolescents with better development of spirituality did not offset the higher initial level of risk and delinquent behavior among those adolescents with a lower level of spirituality (see **Figure 2**). *Post hoc* analyses showed that the adolescents who had better development of moral competence or spirituality always reported lower levels of risk and delinquent behavior as compared with their counterparts who developed badly on these two PYD constructs. Such differences were supported by the significant and negative correlations between moral competence/spirituality and adolescent risk and delinquent behavior across the six waves (see **Table 3**).

### Problem Behavioral Intention Over Time and Its Predictors

The results for the adolescent problem behavioral intention were largely similar to those for adolescent delinquency. First, as shown in **Table 6**, the unconditional quadratic model (Model 3) fitted the data significantly better than did the unconditional linear model (Model 2) [Δχ^2^_(4)_ = 292.57; *p* < 0.001; ΔAIC = 284.57; ΔBIC = 252.96]. The results showed a significant and positive linear slope (β = 0.029, *p* < 0.001) as well as a significant and positive quadratic effect (β = 0.003, *p* < 0.001). As evident in **Figure 1**, the adolescent problem behavioral intention increased over the higher school years and such an increasing trend slightly accelerated over time. Thus, Hypothesis 1b was supported.

The predictive effects were also tested in the intercept, linear and quadratic parameters with the same data analysis procedures used for delinquency. The results in **Table 7** showed that Model 5 including all predictors had the best model fit, compared to Model 3 [Δχ^2^_(15)_ = 211.14; *p* < 0.001; ΔAIC = 181.14; ΔBIC = 67.07] and Model 4 [Δχ^2^_(6)_ = 245.59; *p* < 0.001; ΔAIC = 133.59; ΔBIC = 187.25]. So, the results for the behavioral intention were also interpreted based on Model 5.

The results of Model 5 indicated an increasing trend in the adolescent problem behavioral intention over time (β = 0.041, *p* < 0.001). The results also showed that family economic disadvantage significantly accounted for the variability in the intercept, with the adolescents without economic disadvantage reporting a greater problem behavioral intention. Nevertheless, the *post hoc* analyses revealed that the adolescents with or without family economic disadvantage did not differ significantly in their problem behavioral intention across the six waves (*t*s < 1.75, *p*s > 0.08). Family intactness was also a significant predictor of variability in intercept, with the adolescents living in the non-intact families having a higher initial level of problem behavioral intention. As revealed by the *post hoc* analyses, such a difference reached a significant level at all waves (*t*s > 2.98, *p*s < 0.003). Furthermore, the adolescents with a lower level of moral competence or spirituality demonstrated a greater problem behavioral intention in the initial assessment. Hence, Hypotheses 2c and 2d were also supported.

**FIGURE 3:**
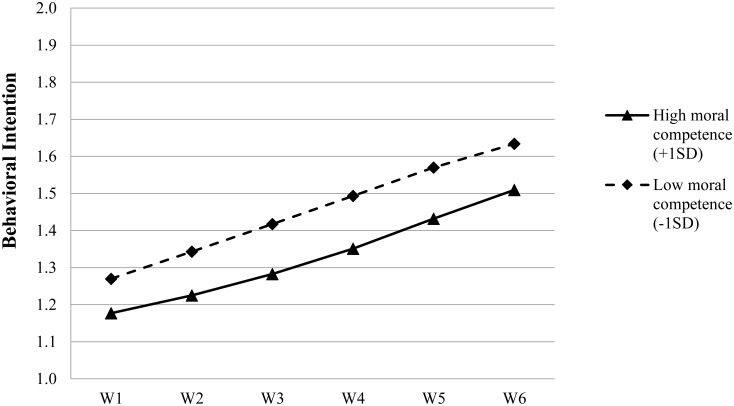
Growth trajectories of adolescent problem behavioral intention as a function of moral competence. The figures were plotted based on Model 5 in **Table 7**. High level indicates 1SD higher than the mean value; low level indicates 1 SD lower than the mean value.

Regarding the linear growth rate, gender, economic disadvantage and moral competence were the three significant predictors of the variability while spirituality was not. Specifically, the growth rate of the problem behavioral intention was slower among females, the adolescents not having family economic disadvantage and the adolescents with a higher level of moral competence (see **Figure 3**). Hence, our Hypothesis 3c was supported while Hypothesis 3d was not. Furthermore, despite a relatively small gamma coefficient, the significant predicting effects of economic disadvantage and moral competence on the quadratic change rate may partially offset their predicting effects on the linear change rate, resulting in not-so-obvious differences in the shape of the change trajectories among the adolescents with or without economic disadvantage, or the adolescents having better or worse development in moral competence (see **Figure 3**).

Similar to delinquency, *post hoc* analyses also indicated that the adolescents who had better development of moral competence or spirituality always reported lower levels of intention to engage in problem behaviors as compared with other adolescents who had lower levels of moral competence or spirituality. Such differences were also reflected by the significant and negative correlations between moral competence/spirituality and the adolescent problem behavioral intention across the six waves (see **Table 3**).

## Discussion

This pioneer study employed two indicators (risk and delinquent behavior, and problem behavioral intention) and examined how they were predicted by moral competence and spirituality in a 6-year longitudinal study. Going beyond the associations between the level of problem behavior and its correlates, the current study also investigated how moral competence and spirituality predicted the developmental trajectories of adolescent problem behaviors. Obviously, the current study enriches our knowledge of Chinese adolescent problem behaviors and their correlates based on a large representative sample in Hong Kong, helping to promote our understanding of the related theoretical issues involved.

As hypothesized, there was a rise in risk and delinquent behavior as well as problem behavioral intention during the secondary school years. The results coincide with the previous findings in the Western societies (Bongers et al., 2003; Deković et al., 2004). While the growth rate of risk and delinquent behavior was gradually slowing down, the rate of change in problem behavioral intention slightly accelerated over time. Such a difference in the growth curves of the two indicators suggested that although adolescent problem behaviors may peak in mid-adolescence, the exact time point may not be identical for the different outcome measures. This finding reflected the complexity of the youth problem behaviors.

Congruent with the previous cross-sectional research in the Western culture (Kim and Brody, 2005; de Kemp et al., 2009), the current study found that the adolescents with a lower level of moral competence defined as virtues reported higher levels of risk and delinquent behavior and intention to engage in problem behavior in future at different time points. In addition to a “snapshot” of the inverse relationship between morality and the adolescents’ problem behaviors found in the cross-sectional research, our study further demonstrated its stability over the secondary school stage. This finding reaffirms the great importance of shaping adolescent moral competence in the high school years when young people restructure the concepts of self and others and start to develop a mature self-identity. According to Hardy et al. (2014), helping adolescents to internalize the social norms and moral principles may enable them to develop good characters and positive moral identities through integrating morality with self-concept.

Similar to moral competence, the results suggested that spirituality is also an important protective factor to lessen the likelihood of problem behaviors for the Chinese adolescents. Theoretically, spirituality defined in terms of the meaning of life helps the adolescents cope with adversity and renders them less likely to externalize the negative life experiences to problem behaviors (Shek, 2012). However, it may be a different case in the Western samples. For example, the Western theorists once suggested that religion may impact on the adolescents earlier while spirituality may play a bigger role during late adolescence and emerging adulthood (Kub and Solari-Twadell, 2013). It is noteworthy that most previous research using the Western samples usually combined spirituality with religiousness due to the high degree of overlap between the two concepts (Yonker et al., 2012), although the studies showed different influences of the two concepts (Salas-Wright et al., 2013).

Noteworthy, the present study also found that students without economic disadvantage or living in intact families demonstrated lower level and slower growth rate in externalizing problems, suggesting that both adolescents’ internal assets (e.g., moral competence and spirituality) and external assets serve as protective factors. Moral competence in the scope of Chinese virtues may imply one’s self-regulation that promote one’s internal motivation to behave in a morally and socially acceptable manner. One can argue that this type of self-regulation not only suggests one’s ability to exercise self-control, but also reduces the likelihood that adolescents treat misconduct as an alternative for action, both of which would significantly lower the risk of problem behaviors (Wikström and Treiber, 2007; Svensson et al., 2010). Referring to the theory of self-regulated and external-regulated learning and other behaviors (de la Fuente-Arias, 2017), while adolescents’ internal assets (e.g., moral competence) promote self-regulated moral behaviors, familial assets as external-regulators reinforce adolescent personal-regulation.

The present study also shed light on the predicting effects of the two developmental assets on the changes of problem behaviors over time. For risk and delinquent behavior, while moral competence did not show a significant effect on its linear change rate, a higher level of spirituality was associated with a *faster* linear increasing rate. For problem behavioral intention, a higher level of moral competence predicted a slower linear growth rate, whereas spirituality did not significantly predict the growth rate. There are two possible explanations for the insignificant predicting effects of moral competence on delinquency growth rate and of spirituality on problem behavioral intention growth rate. First, with risk and delinquent behavior gradually reaching its maximum level, the predicting effects of the developmental assets on the growth rate may confront a ceiling effect. This mechanism may also explain the prior findings that the predicting effects of morality on delinquency were rather small and would decline during middle and late adolescence (Matsueda, 1989; Raaijmakers et al., 2005). Second, given that the problem behavioral intention in the present study was at an early developmental stage featured with an overall low level, the floor effect may operate on the predicting effects of the developmental assets.

There may be other underlying mechanisms coexisting with the ceiling effect or floor effect. For risk and delinquent behavior, the positive association between its growth rate and spirituality was in line with the previous findings that the adolescents with better overall PYD had a *faster* increasing rate of delinquent behavior (Shek and Lin, 2016b). As suggested by Shek and Lin (2016b), the better-developed adolescents were more motivated to “experiment” on the “*safe*” risk acts. This might be valid as the present study found the high occurrences of those “*safe*” misconducts such as cheating and speaking foul language. However, such a mechanism may not work for the problem behavioral intention assessed in the present study, as this indicator included activities that are not “*safe*,” such as drinking and drug abuse, which may cause severe damage to one’s health and may be perceived as seriously wrong by the Chinese adolescents (Tisak et al., 2017). Instead, the negative relationship between moral competence and the linear change rate of the problem behavioral intention echoes the common PYD reasoning that better development of personal assets builds a constructive groundwork for adolescent behavioral adjustment (Lerner et al., 2013; Shek and Leung, 2013). Nevertheless, the *faster* increasing rate of risk and delinquent behavior among the adolescents with high spirituality was a novel finding which should be further addressed in future studies. Moreover, it should be noted that consistent with the prediction of the PYD literature, the levels of problem behaviors were consistently lower in the groups with higher spirituality.

Based on the current findings, it can be conjectured that different underlying mechanisms may operate at different developmental stages of problem behaviors, and the impact patterns may differ between different psychosocial competencies. While the present findings are pioneer, it is suggested that with more waves of investigation covering a longer period (e.g., from late childhood to emerging adulthood) or with different age cohorts being longitudinally assessed simultaneously, different predictive effects of one developmental asset at different developmental stages of one problem behavior could be uncovered. For example, Raaijmakers et al. (2005) reported that the longitudinal relationship between morality and delinquency followed different pathways among three age cohorts. Besides, more types of developmental assets should be examined simultaneously to enable the comparison of their relative effects.

Practically speaking, the present findings provide exciting evidence for enhancing adolescent health functioning and preventing the youth problem behaviors through promoting moral competence and/or spirituality. In fact, such programs have already been successfully implemented in the Western countries. For example, one international spirituality program entitled “All Round Training in Excellence Program” involved the adolescents from London, Vancouver, Johannesburg, and Mumbai, and the program was regarded as having positive influence on the participants’ mental health and well-being (Pandya, 2015). In addition, the PYD programs promoting moral competence, spirituality and other psychosocial competencies were perceived as effective in preventing problem behaviors and promoting health development in children and adolescents (Catalano et al., 2002, 2012). These programs seem warranted for the Chinese adolescents as well, given the worrying increasing trend of the adolescents’ problem behavior and the dearth of PYD programs attempting to nurture moral competence, spirituality and other developmental assets in the Chinese adolescents (Shek and Yu, 2011; Shek and Leung, 2013).

The present study has several limitations. First, participant attrition is non-random with boys, those with externalizing problem behaviors or lower level of developmental assets dropping out more frequently. This systematic attrition may cause bias to the research findings. Fortunately, we imputed the missing values of the core research variables using multiple imputation and the results showed that the findings obtained from the imputed data and original data were almost identical. This finding suggested that the non-random attrition did not significantly affect the present findings. Nevertheless, further research could try to retain the participants and avoid the systematic attrition problem in the first place.

Second, although the data of moral competence and spirituality were collected in each wave, only the initial levels of moral competence and spirituality were used to predict the occurrence and the change in adolescents’ problem behaviors. We only controlled the initial status of socio-demographic variables and did not consider the possibility that the participants’ economic condition and family structure might have changed across the 6 years. In addition, we measured competence and spirituality with shortened scales, which may cause content validity problems, although a usual procedure was utilized to select items and reliabilities of the two shortened scales were good. Therefore, future research could consider replicating the present findings by using the full scales.

Third, although most studies including the present one assumed the influence of developmental assets on adolescent problem behaviors, it is possible that the relationship between adolescent competence (e.g., moral competence) and problem behaviors is bidirectional in nature (Raaijmakers et al., 2005; Arbeit et al., 2014). Knowing that not addressing the reciprocity of the effects within a limited length is one limitation, we have been attempting to fill this research gap by examining the reciprocal relationship between adolescent moral competence in terms of virtues and externalizing problems in the next paper (Shek and Zhu, unpublished). Different from the current paper which used 6-wave data and individual growth curve modeling, the next paper will focus on three selected time points during early adolescence and use cross-lagged analyses.

Fourth, the data on moral competence, spirituality and problem behaviors were based on the self-report measures. The self-reported problem behaviors were regarded as efficient, precise, reliable and valid in longitudinal studies (Emler and Tarry, 2007). It is also a common practice to measure positive traits by self-report inventories (Park and Peterson, 2006; Shek et al., 2007). However, the participants might provide socially desired answers by under-reporting their problem behaviors or over-estimating their moral competence and spirituality. Given that the instructions of not communicating with classmates and emphasis on anonymity were made very clearly before questionnaire administration in each wave, social desirability bias and peer pressure might have been reduced. Hence, it would be helpful to use social desirability measures or obtain the data from other informants such as teachers and parents (de Kemp et al., 2009). Nevertheless, considering the ethical and practical concerns (e.g., the adolescents themselves know the most of their own lives and experiences), the self-report measures are most appropriate and widely used in adolescent research (e.g., Thornberry and Krohn, 2003).

Fifth, as the present study only examined two developmental assets, future studies may extend the present findings by investigating and comparing the effects of more developmental assets. In addition, within the scope of morality, there are also other constructs, such as moral beliefs and moral emotions (e.g., shame and guilty), which also serve as protective factors for reducing adolescent delinquency (Krettenauer and Eichler, 2006; Beerthuizen and Brugman, 2016; Piquero et al., 2016). It is possible that these constructs have interactions with moral competence, which need to be addressed in future research.

Last, problem behaviors were considered as the overall risk and delinquent behavior and intention to engage in problem behaviors, limiting the present findings to the overall level of problem behaviors. Concerning the not-particularly high reliability of measuring scales and the prevalence of different forms of risk and delinquent behaviors observed in the present study, it is possible that some behaviors are more common (e.g., speaking foul language) than others (e.g., gang fighting). However, as stated in the section of results, an examination of the inter-item correlation coefficients showed that they are acceptable. Nevertheless, it would be insightful to examine each form of risk and delinquent acts separately or compare risk behavior with delinquency that presumes illegal acts in the future research.

In short, the present study is the first longitudinal study in different Chinese societies which examined this issue in the high school years using the individual growth curve modeling. Second, the findings suggested that there is a need to differentiate between the levels and trajectories of adolescent problem behaviors. Third, the present findings suggested that moral competence and spirituality can function as important protective factors for lowering the level of problem behaviors among Chinese adolescents.

## Ethics Statement

The project was approved by the Human Subjects Ethics Sub-committee (HSESC) (or its Delegate) of the authors’ university. The participating schools and students’ parents were well informed that the data collected from the students would be kept confidential and used only for research purposes. Their written consent was obtained. Before each wave of data collection, the principles of voluntary participation and confidentiality were explained to the participating students and their written consent was also obtained.

## Author Contributions

DS designed the project and contributed to all steps of the work. XZ contributed to the idea construction and data interpretation of the work, drafted the work and revised it based on the critical comments provided by DS. All authors approved of the final version of the manuscript and agree to be accountable for all aspects of the work in ensuring that questions related to the accuracy or integrity of any part of the work are appropriately investigated and resolved.

## Conflict of Interest Statement

The authors declare that the research was conducted in the absence of any commercial or financial relationships that could be construed as a potential conflict of interest.
